# Effects of remote digital monitoring on oral hygiene of orthodontic patients: a prospective study

**DOI:** 10.1186/s12903-021-01793-9

**Published:** 2021-09-07

**Authors:** Linda Sangalli, Fabio Savoldi, Domenico Dalessandri, Stefano Bonetti, Min Gu, Alberto Signoroni, Corrado Paganelli

**Affiliations:** 1 Department of Information Engineering, Faculty of Engineering, University of Brescia, Via Branze 38, 25123 Brescia, Italy; 2 Division of Orofacial Pain, College of Dentistry, University of Kentucky, 740 S. Limestone, Lexington, KY 40536 USA; 3 Division of Paediatric Dentistry and Orthodontics, Faculty of Dentistry, 2/F, Prince Philip Dental Hospital, The University of Hong Kong, 34 Hospital Road, Sai Ying Pun, Hong Kong SAR; 4 Dental School, Department of Medical and Surgical Specialties, Radiological Sciences and Public Health, University of Brescia, Piazzale Spedali Civili 1, 25123 Brescia, Italy

**Keywords:** Oral hygiene, Telemonitoring, Dental Monitoring, Orthodontics, Digital dentistry, COVID-19

## Abstract

**Background:**

Remote digital monitoring during orthodontic treatment can help patients in improving their oral hygiene performance and reducing the number of appointments due to emergency reasons, especially in time of COVID-19 pandemic where non-urgent appointments might be discouraged.

**Methods:**

Thirty patients scheduled to start an orthodontic treatment were divided into two groups of fifteen. Compared to controls, study group patients were provided with scan box and cheek retractor (Dental Monitoring®) and were instructed to take monthly intra-oral scans. Plaque Index (PI), Gingival Index (GI), and White Spot Lesions (WSL) were recorded for both groups at baseline (t_0_), every month for the first 3 months (t_1_, t_2_, t_3_), and at 6 months (t_4_). Carious Lesions Onset (CLO) and Emergency Appointments (EA) were also recorded during the observation period. Inter-group differences were assessed with Student's *t* test and Chi-square test, intra-group differences were assessed with Cochran’s Q-test (significance α = 0.05).

**Results:**

Study group patients showed a significant improvement in plaque control at t_3_ (*p* = 0.010) and t_4_ (*p* = 0.039), compared to control group. No significant difference was observed in the number of WSL between the two groups. No cavities were detected in the study group, while five CLO were diagnosed in the control group (*p* = 0.049). A decreased number of EA was observed in the study group, but the difference was not significant.

**Conclusions:**

Integration of a remote monitoring system during orthodontic treatment was effective in improving plaque control and reducing carious lesions onset. The present findings encourage orthodontists to consider this technology to help maintaining optimal oral health of patients, especially in times of health emergency crisis.

## Background

Oral hygiene should be routinely controlled in patients undergoing orthodontic treatment. Several studies have demonstrated a rapid decline in the level of oral hygiene status after the initial bonding of the orthodontic appliance [1], which constitutes an obstacle to the oral hygiene procedures [2] and may lead to changes in composition of the bacterial flora [3]. Presence of dental plaque on the tooth surface for a critical length of time is associated with increased chance of demineralization and white spot lesions [4], along with gingival inflammation [4]. This can negatively affect the clinical outcome due to possible discontinuation of the orthodontic treatment [4]. Moreover, teenagers may be at high risk of carious lesion onset because of lack of cooperation and difficulties during the daily oral hygiene procedures [5].

Nevertheless, oral hygiene status can significantly ameliorate with reward systems or active reminder tools, especially when teenagers are engaged by technological supports [6, 7]. Two systematic reviews have shown a positive influence of text messages on behavioural changes [8], and a significant association between the use of mobile technologies and the improvement in dental plaque control and gingival bleeding [9]. Previous studies proposed a system of active reminders for adolescents undergoing orthodontic treatment by weekly text messaging their parents [8, 10–12], using WhatsApp chat room for sharing “selfies” of their smile [13], and using computer-based training to teach Fones brushing technique or modified Bass technique [14]. In addition, frequently used social media among young subjects, such as Instagram [15], or other digital platforms, such as YouTube [16], have shown to improve oral health knowledge among orthodontic patients. Still, some studies found no positive effect of instructions about oral hygiene during orthodontic treatment by using social media-based and messaging apps, respectively [17, 18].

A recent new tool for remote monitoring is Dental Monitoring**®** (DM, Paris, France), a software-based program that allows patients to capture their occlusion using a smart phone and a scan box. It consists of three integrated platforms: a mobile app for the user, a movement-tracking algorithm, and a web-based Doctor Dashboard®, where the clinician can check the treatment progress, teeth movement, integrity of appliances, and oral hygiene status through the analysis of pictures that are periodically taken by the patient [19]. Such remote monitoring is especially important in times of COVID-19 pandemic, as it allows maintaining continuity of care, while minimizing the risk of disease transmission and optimizing the use of resources [20].

To the best of our knowledge, the present work may be the first investigation of the oral hygiene status of orthodontic patients using the scan box. The aim of the study was to verify whether an active reminder—such as DM—integrated to the traditional orthodontic standard of care, could help patients in maintaining a better oral hygiene during the first six months of treatment, and in reducing the number of appointments due to emergency reasons.

## Methods

### Study subjects

Setting a clinically significant difference of 0.5 points in the Plaque Index (PI) between the two groups, a Standard Deviation (SD) of 0.5 based on a previous study [21], a significance level α = 0.05, and a power beta = 80 %, the required sample size was calculated as 17 subjects for each group [22]. Considering the drop-out rate, forty consecutive patients scheduled to start an orthodontic treatment between January and December 2018 were proposed to participate in the study. Eight of them deviated from the inclusion criteria during the study and two eventually declined to participate. Inclusion criteria were to be online daily, to have access to a smartphone, and to undergo a non-extraction orthodontic treatment with fixed brackets or aligners. Exclusion criteria were a daily supplemental fluoride regimen, and physical or cognitive disabilities impeding to take pictures or to perform oral hygiene procedures.

Among the thirty participants enrolled in the study (mean age 20.6 ± 9.0 years), 15 patients (7 males and 8 females, mean age 24.9 ± 10.9 years) were assigned to the study group: 5 patients were treated with fixed buccal multi-bracket appliance, and 10 patients with aligners. The control group (15 patients, 7 males and 8 females, mean age 16.3 ± 3.2 years) consisted of 11 patients treated with fixed buccal multi-bracket appliance, 3 patients with aligners, and 1 patient with fixed lingual multi-bracket appliance.

The study was performed in accordance with the ethical standards as laid down in the Declaration of Helsinki and was approved by the Institutional Review Boards of the University of Brescia.

### Treatment protocol

Both groups were treated by the same orthodontist (L.S.). The fixed buccal multi-brackets were self-ligating Empower® brackets with MBT prescription and 0.022-inch slot (American Orthodontics, Sheboygan, WI), applied with direct bonding technique. Patients undergoing treatment with aligners received Invisalign® appliances (Align Technology, San Jose, CA). The fixed lingual multi-bracket appliance was Win® (DW Lingual Systems, Bad Essen, Germany), applied with indirect bonding technique.

### Oral hygiene protocol and assessment

An oral hygiene kit, containing toothbrush and toothpaste (Mentadent**®**, Unilever, the Netherlands), a mouthwash and a dental floss (GUM**®**, Sunstar Suisse, Switzerland), and an inter-proximal aid (Krugg, Melville, New York, USA) were provided to each patient, with the instructions to brush their teeth at least twice per day and floss once per day. The patients and their parents were educated by the clinician during the first in-person visit, also regarding the importance of limiting sugar consumption and avoiding sticky food.

During the appointment of the bonding of the appliance or of the delivery of the aligners (t_0_), every month for the first three months (t_1_, t_2_, t_3_) and at 6 months (t_4_), Plaque Index (PI), Gingival Index (GI), and White Spot Lesions (WSL) were clinically assessed [23].

At chair-side visits, PI was scored by evaluating the presence of plaque on the mesial, buccal, distal and lingual surfaces of 1.6, 1.4, 2.1, 4.6, 3.2, 3.5, assigning a score from 0 to 3 for each surface and calculating the mean value (Table 1) [24]. GI was scored by evaluating the presence of inflammation on the buccal surfaces of 1.6, 1.4, 2.1, 4.6, 3.2, 3.5, assigning a score from 0 to 3 and calculating the mean value (Table 1) [24]. To evaluate WSL, teeth were air-dried for 5 s and then the buccal surface close to the gingival contour was evaluated, assigning a score from 0 to 3 (Table 1). [24].

**Table 1:** Plaque Index, Gingival Index and White Spot Lesions scales used for clinical evaluation

Plaque Index (PI) score	
0	Absence of plaque in the gingival area
1	Slight deposit of plaque at gingival margin
2	Moderate accumulation of soft deposits covering less than half of the surface
3	Abundance of deposits covering more than half of the surface
Gingival Index (GI) score	
0	Normal gingiva, no inflammation, bleeding or swelling
1	Mild inflammation, slight edema and color change; no bleeding
2	Moderate inflammation, redness, swelling; bleeding when probing
3	Important inflammation, marked redness and edema; spontaneous bleeding
White Spot Lesions (WSL) score	
0	No visible WSL or surface disruption (no demineralization)
1	Visible WSL without enamel surface disruption (mild demineralization)
2	Visible WSL with roughened surface (moderate demineralization)
3	Visible WSL requiring restoration (severe demineralization)

*PI* Plaque Index; *GI* Gingival Index; *WSL* White Spot Lesions

For both groups, the number of Emergency Appointment (EA) and Carious Lesions Onset (CLO) were recorded during the observation period. At each visit, both groups were additionally monitored using plaque-disclosing tablets (Red-Cote®, GUM, Sunstar Suisse, Switzerland). The outcome was photographed and shown to the patient.

**Fig. 1:**
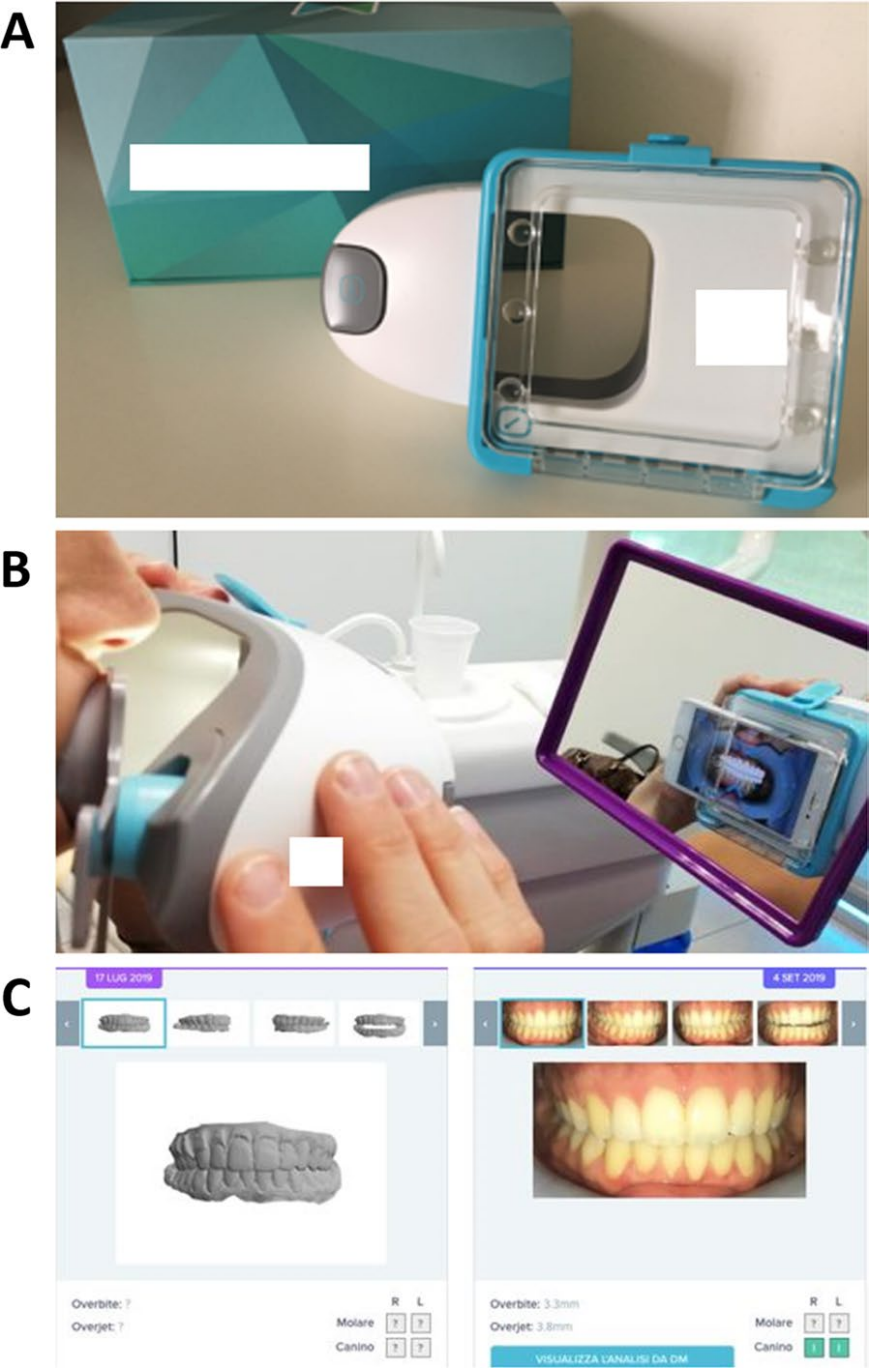
Scan box for remote 2D photo monitoring by Dental Monitoring® (**A**), device used by the patient (**B**), and software interface (**C**)

In addition to the chair-side appointments, the study group was also monitored with remote 2D photo monitoring (Dental Monitoring**®**, DM, Paris, France) (Fig. 1). At baseline, the study group patients were asked to download the DM app, and were instructed to take pictures of their mouth to be uploaded. A scan box and a dedicated cheek retractor by DM were provided to each patient of the study group. The first scan was made together with the orthodontist to ensure proper use of the device. The frequency of scans was monthly, and DM evaluated the pictures uploaded by the patient upon the oral hygiene status, checking the periodontal gingival health, the amount of plaque left on the teeth, and sent a text message to the patient with its evaluation.

### Data analysis

The normality of the data distribution was verified with Shapiro–Wilk test. Differences between groups regarding PI, GI, and WSL were assessed by Student's *t*-test. Intra-group differences of PI, GI, and WSL at different time-points were assessed using Cochran’s Q-test. Chi-Square test was used to compare CLO and EA between the two groups. Statistical analysis was performed with statistical software (SPSS^©^ Statistics 27, IBM, Armonk, NY, USA) at significance level α = 0.05.

## Results

### Intra-group differences

The intra-group differences at different time-points for study group patients were significant for PI, GI and WSL (Table 2). PI values decreased from 0.51 (± 0.45) at t_0_, to 0.31 (± 0.43) at t_4_ (*p* < 0.001). GI values decreased from 0.88 (± 0.52) at t_0_, to 0.36 (± 0.42) at t_4_ (*p* < 0.001). WSL values decreased from 1.10 (± 1.50) at t_0_, to 0.80 (± 1.40) at t_4_ (*p* = 0.035).

**Table 2:** Mean values at time-points t_0_ (baseline), t_1_ (1 month), t_2_ (2 months), t_3_ (3 months), and t_4_ (6 months) of Plaque Index, Gingival Index, and White Spot Lesions for the study group and control group

	PI						GI						WSL					
	Study group	SD	Control group	SD	Delta	*p*-value*	Study group	SD	Control group	SD	Delta	*p*-value*	Study group	SD	Control group	SD	Delta	*p*-value*
t_0_	0.51	0.45	0.44	0.47	− 0.07	0.383	0.88	0.52	0.43	0.43	− 0.45	**0.013**	1.10	1.50	1.13	2.06	0.29	0.464
t_1_	0.41	0.43	0.63	0.53	0.22	0.069	0.61	0.56	0.48	0.45	− 0.13	0.259	1.10	1.90	0.93	2.08	− 0.17	0.434
t_2_	0.42	0.42	0.69	0.54	0.27	0.050	0.45	0.45	0.58	0.48	0.13	0.281	0.73	1.02	1.00	1.90	0.27	0.372
t_3_	0.35	0.42	0.74	0.48	0.39	**0.010**	0.43	0.42	0.56	0.45	0.13	0.198	0.66	1.10	0.71	1.50	0.04	0.500
t_4_	0.31	0.43	0.56	0.43	0.25	**0.039**	0.36	0.42	0.47	0.35	0.11	0.189	0.80	1.40	0.93	1.70	0.13	0.458
*p*-value^#^	** < 0.001**		** < 0.001**				** < 0.001**		** < 0.001**				**0.035**		0.085			

Intergroup differences at each time-point, and intra-group differences at different time-points are reported. Delta represents the value of study group minus the value of control group

*PI* Plaque Index; *GI* Gingival Index; *WSL* White Spot Lesions; *SD* Standard Deviation

Statistically significant *p*-values are reported in bold

^#^Cochran’s Q-test; *Student’s *t*-test

In the control group, PI and GI values increased significantly, while WSL values did not reveal a significant change (Table 2). PI values increased from 0.44 (± 0.47) at t_0_, to 0.56 (± 0.43) at t_4_ (*p* < 0.001). GI values increased from 0.43 (± 0.43) at t_0_, to 0.47 (± 0.35) at t_4_ (*p* < 0.001).

### Inter-group differences

**Fig. 2:**
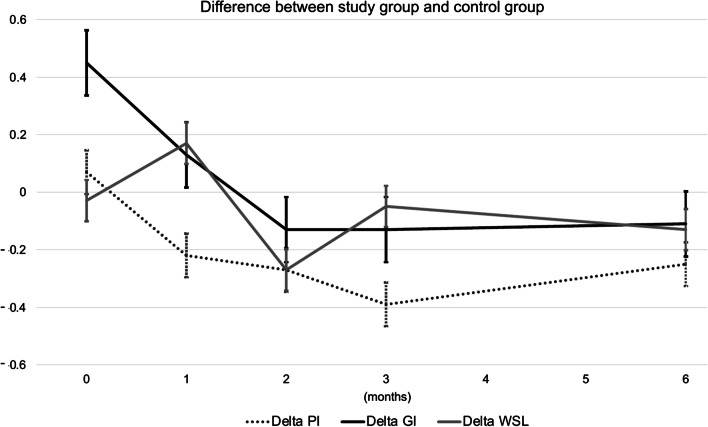
Difference between study group and control group regarding Plaque Index (PI), Gingival Index (GI), and White Spot Lesions (WSL) at different time-points. Data were recorded at baseline (t_0_), after one month (t_1_), two months (t_2_), three months (t_3_), and six months (t_4_). The graph shows a tendency among the study group patients to progressively exhibit a better PI and GI compared to the control group. However, no evident differences were present in terms of WSL

Regarding PI, despite the two groups had similar values at baseline, at t_3_ the mean value of the study group was significantly lower (0.35 ± 0.42) compared to the control group (0.74 ± 0.48) (*p* = 0.010). At t_4_, the mean value of the study group was also significantly lower (0.31 ± 0.43) compared to the control group (0.56 ± 0.43) (*p* = 0.039) (Table 2 and Fig. 2).

With regard to GI, even though at baseline the mean value for the study group was higher (0.88 ± 0.52) compared to the control group (0.43 ± 0.43) (*p* = 0.013), the two groups reached similar values during the observation period (Table 2 and Fig. 2).

The difference between the two groups with regard to WSL was not significant at any timepoint (Table 2 and Fig. 2).

As for CLO, no cavities were detected in the study group, while five CLO were diagnosed in the control group (*p* = 0.049).

With regard to EA, although the patients of the control group recorded more extra appointments (7.0) than those of the study group (3.9), the difference was not statistically significant.

## Discussion

Systems of tele monitoring have become one of the most widespread response of the medical field to the current COVID-19 pandemic [25] and—in appropriate cases—have shown to be as effective and well-accepted by patients as the standard care of treatment [26]. Remote monitoring systems are part of Artificial Intelligence Driven Remote Monitoring (AIRM) [27]. Several clinical applications of these technologies in orthodontics include monitoring the integrity and side effects of the appliance, the gingival health of the patients, and the loss of tracking of the dental movements obtained with aligners [28].

The present study focused on the monitoring of orthodontic patients, and the record of PI, originally described by Silness and Loe [24], was one of the parameters selected to evaluate the level of their oral hygiene status. In the literature, the PI was used in the majority of the trials [29], as it allows a rapid assessment and it is workable in dental offices without expensive costs. According to best clinical practice principles, plaque assessment further included the use of disclosing-plaque tablets, which evidence was photographed and shown to patients and parents in order to enhance collaboration and oral hygiene independently from the use of remote digital monitoring. In fact, it is an affordable and easy-to-perform visual method that provides a rapid feedback to improve brushing technique and conscious awareness [30]. With regard to PI, the control group showed a worsening of the oral hygiene level, although after three months the accumulation of plaque substantially decreased. This might be interpreted as a sign that instructions and visual method of disclosing-plaque tablets eventually enhanced awareness in patients and families on the importance of a good oral hygiene [31]. Yet, the final PI value at six months remained higher than the value at baseline, confirming the difficulty of patients in keeping teeth clean during orthodontic treatment [32], which may be especially difficult in case of fixed appliances with complex design [33]. As for the study group, PI values steadily decreased over time, and after six months the plaque detected was less than the initial value. Overall, the trend shown by the two groups was the opposite, in accordance with the literature [34]. Accordingly, the differences between control group and study group regarding PI were significant during the latest assessments at three and six months.

With regard to GI, the control group showed a worsening of the periodontal health status during the first months of treatment, according to the literature [30]. Conversely, in the study group, the GI dropped by more than half of the initial values by the end of the observation period. However, the difference between the two groups was not significant and it is unclear whether DM also helped to improve the periodontal status.

Concerning WSL, their onset steadily decreased over time in the study group. It might be possible that implements aimed to improve oral hygiene also helped in increasing cooperation of patients, in accordance with a systematic review [31]. Still, the difference between control group and study group was not significant and the present study was inconclusive in showing improvements of this aspect when DM was used.

A relevant finding was the difference in terms of CLO between the two groups, where DM is likely to have played an important role in enhancing the attention of the patients on oral hygiene control. Still, the overall improvement in oral hygiene of the study group might be partly due to the Hawthorne Effect [35], as patients in the study group were aware of being under monitoring by the examiner.

According to the literature, monitoring the oral hygiene status of orthodontic patients may decrease the number of EA [36, 37]. The present study confirmed a similar trend, even though the reduction of extra appointments in the study group was not statistically significant.

Nevertheless, such remote digital technologies may include potential concerns, including a possible deterioration in patient-clinician relationship due to a reduced number of in-person appointments, and the inevitable cost of using AIRM itself [28]. Considering the novelty of such technological advances and the lack of well-defined standards [27], the clinicians should carefully balance the benefits of in-office visits with the advantages of remote monitoring, while maintaining standard of care.

### Limitations

The value of GI was significantly different at baseline between study group and control group, and randomized studies are necessary to confirm the present findings. Further works may also extend the observation period to one year, in order to complete the orthodontic treatment, or longer, to observe the retention period as well. In the present study, patients used DM dedicated cheek retractors when taking the scans. However, every person is unique in the amount of maximal mouth opening and cheek muscle tonicity, which may have affected the tooth visibility in the oral cavity. Moreover, the scans of two 10-year-old patients were often rejected by the software, maybe due to the poor manual skills of such young subjects. Thus, how well teeth are captured may vary depending on the manual skills of each individual, and such variation may have influenced the present results.

## Conclusions

Remote monitoring applied during orthodontic treatment showed encouraging results in reducing plaque and onset of carious lesions. However, incidence of emergency appointments, gingival status, and onset of white spot lesions may not significantly improve. These preliminary results suggest potential application of this technology in clinical practice, especially in times when routine clinical check-ups might be compromised. Further randomised studies including larger and more homogeneous groups of participants are advisable to confirm the present findings.

## Data Availability

The datasets generated and analyzed during the current study are not publicly available due to privacy reasons (images of patients), but are available from the corresponding author on reasonable request.

## Abbreviations

DM: Dental Monitoring
PI: Plaque Index
GI: Gingival Index
WSL: White Spot Lesions
CLO: Carious Lesion Onset
EA: Emergency Appointments
COVID-19: Coronavirus disease 2019
AIDRM: Artificial intelligence driven remote monitoring
